# Long Non-coding RNAs LINC01679 as a Competitive Endogenous RNAs Inhibits the Development and Progression of Prostate Cancer via Regulating the miR-3150a-3p/SLC17A9 Axis

**DOI:** 10.3389/fcell.2021.737812

**Published:** 2021-11-25

**Authors:** Yuan-yuan Mi, Chuan-yu Sun, Li-feng Zhang, Jun Wang, Hong-bao Shao, Feng Qin, Guo-wei Xia, Li-jie Zhu

**Affiliations:** ^1^Department of Urology, Affiliated Hospital of Jiangnan University, Wuxi, China; ^2^Department of Urology, Huashan Hospital, Fudan University, Shanghai, China; ^3^Department of Urology, Affiliated Changzhou No. 2 People’s Hospital of Nanjing Medical University, Changzhou, China

**Keywords:** LINC01679, miR-3150a-3p, SLC17A9, prostate cancer, ceRNA

## Abstract

Long non-coding RNAs (lncRNAs) have been indicated as the candidate factors to predict cancer prognosis. However, it is still unknown whether lncRNA combinations may be utilized for predicting overall survival (OS) of prostate cancer (PCa). The present work focused on selecting the potent OS-related lncRNA signature for PCa and studying its molecular mechanism to enhance the prognosis prediction accuracy. Differentially expressed lncRNAs (DElncRNAs) or differentially expressed genes (DEGs) were obtained based on TCGA database by R software “edgeR” package. lncRNAs or mRNAs significantly related to PCa were screened through univariate as well as multivariate Cox regression, for the construction of the risk model for prognosis prediction. Moreover, this constructed risk model was validated through ROC analysis, univariate regression, and Kaplan–Meier (KM) analysis. Additionally, we built a lncRNA–miRNA–mRNA ceRNA network through bioinformatics analysis. Colony formation, CCK-8, flow cytometry, scratch, and Transwell assays were performed based on PCa cells subjected to small interfering RNA (siRNA) targeting LINC01679/SLC17A9 and vector expressing LINC01679/SLC17A9 transfection. Thereafter, the ceRNA mechanism was clarified *via* qRT-PCR, Western blotting (WB), RNA pull-down, and luciferase reporter assays. Nude mouse tumor xenograft was established to examine LINC01679’s oncogenicity within PCa cells. According to our results, LINC01679 depletion promoted cell proliferation, metastasis, tumor growth, and inhibited cell apoptosis *in vivo* and *in vitro*, which was also associated with poor survival. LINC01679 regulated miR-3150a-3p level by sponging it. Importantly, miR-3150a-3p overexpression was related to the increased proliferation and decreased apoptosis of PCa cells. Rescue assays suggested that miR-3150a-3p mimics rescued the repression on PCa progression mediated by LINC01679 upregulation, but SLC17A9 downregulation reversed the miR-3150a-3p inhibitor-mediated repression on PC progression. Importantly, SLC17A9 downregulation rescued the repression on PCa progression mediated by LINC01679 upregulation. LINC01679 and SLC17A9 are tightly associated with certain clinicopathological characteristics of PCa and its prognostic outcome. In addition, LINC01679 is the ceRNA that suppresses PCa development through modulating the miR-3150a-3p/SLC17A9 axis.

## Introduction

Prostate cancer (PCa) is the most common reproductive cancer in men. PCa ranks the second and fifth in terms of its morbidity and death-related cause among men worldwide (Wang G. et al., 2018). The occurrence and development of PCa is not achieved overnight; instead, it needs to go through a long evolutionary cycle. Generally, genomic mutations, changes in the cellular ecosystem, unhealthy living habits, and living environment can lead to the occurrence of PCa (Grozescu and Popa, 2017). Noteworthily, the transformation from normal prostate cells to tumor cells and further invasion and metastasis are a highly heterogeneous and extremely complex process (Chang et al., 2014). Therefore, in view of different development links, it is of important theoretical and clinical significance to use big data, cloud computing technology, and translational biomedical informatics methods to achieve accurate intervention, slow down, or even reverse the process of prostate cancerization through systematic analysis, integration, and identification of key elements. The interactions between biomolecules form the large-scale and complex biomolecule networks, including the protein–protein interaction (PPI) network (Athanasios et al., 2017), gene co-expression network (van Dam et al., 2018), miRNA–mRNA regulatory network (Lou et al., 2019), and ceRNA regulatory network (Jiang et al., 2020). Analyzing network structure and function is of crucial significance to understand complex biological problems. Therefore, it has always been an important issue in network science and systems biology to find the key sites or key role relationships in the network system, so as to measure or evaluate the stability of biological systems. Long non-coding RNAs (lncRNAs) are suggested in studies as the oncogenes or tumor suppressors for cancer (Wang J. et al., 2018; Wang et al., 2019; Zhuang et al., 2019). Competitive endogenous RNAs (ceRNAs), including mRNAs, pseudogenes, lncRNAs, and circRNAs, can bind competitively to miRNAs, thereby affecting miRNAs’ effect on target genes (Qi et al., 2015). This study built a ceRNA network to find key sites affecting the stability of the network and system and their relationship, so as to provide theoretical reference for diagnosing and treating PCa and other cancers.

Long non-coding RNAs are RNAs that are more than 200 nucleotides (nt) long, which cannot encode proteins (Wang J. et al., 2018). Some lncRNAs can serve as the oncogenes or tumor suppressor genes of PCa (Lingadahalli et al., 2018; Shang et al., 2019; Wu M. et al., 2019). Their abnormal expression is closely related to PCa genesis and progression, which are the markers for diagnosing and targets for treating PCa. PCA3, an early discovered lncRNA molecule, is reported to be related to PCa (Soares et al., 2019). Its expression is prostate-specific and can be used in the clinical diagnosis and management of PCa. In PCa, PCAT1 can negatively regulate the functional defect of BRCA2-induced homologous recombination and promote PCa cell growth through increasing cMyc; as a result, it can be used as a prognostic marker of PCa (Prensner et al., 2014). At present, integrated analysis based on ceRNA competition mechanism is the mainstream method to identify the lncRNA markers for cancer. Many articles suggest that lncRNAs play the role of ceRNAs for regulating PCa development (Wu X. et al., 2019; Zhang Y. et al., 2019). Notably, lncRNA UCA1 is a ceRNA that can enhance PCa development through the sponge of miR-143 (Yu et al., 2020). lncRNA HCP5 enhances PCa cell growth through sponging miR-4656 for regulating CEMIP level (Hu and Lu, 2020). Moreover, after being activated by RAX5, lncRNA FOXP4-AS1 enhances PCa proliferation through the sequestration of miR-3184-5p for upregulating FOXP4 (Wu X. et al., 2019). However, the function of LINC01679 in tumor progression has not been studied yet.

This work focused on the role of LINC01679 in PCa and the related molecular mechanism. It was found that LINC01679 inhibited PCa genesis and development through sponging miR-3150a-3p and specifically targeting SLC17A9. Findings from this study help to diagnose and treat PCa.

## Materials and Methods

### Patient Datasets and Processing of Long Non-coding RNAs and mRNAs

All data were obtained based on the TCGA database. We adopted the Data Transfer Tool (GDC Apps) to download clinical and gene expression profiling data from PCa cases^1^. Altogether, 540 PCa cases were later randomized as training and validation sets (ratio, 7:3) to carry out integrated analysis by the ‘‘caret’’ package. The sample inclusion criteria in both sets were as follows: (1) samples were randomized as training or test set; (2) comparable clinical characteristics of samples from both sets. All data were freely accessible, and approval by Ethics Committee was unnecessary. All data were processed according to relevant NIH TCGA human subject protection policies and data access policy^2^.

The Illumina HiSeq RNASeq platform was adopted to obtain mRNA or lncRNA expression profiles in PCa cases, which were subsequently normalized according to TCGA. Thereafter, the “edgeR” function of the R package was utilized for detecting differentially expressed RNAs (DERNAs) and identifying differentially expressed lncRNAs (DElncRNAs) by adopting the thresholds of adjusted *p* < 0.05 and log2 fold change (FC) > 2.0.

### Construction of the Long Non-coding RNAs or mRNA Signature

The associations between mRNA or lncRNA levels and patient overall survival (OS) were analyzed by the univariate Cox model. At the same time, univariate analysis was conducted to identify significant lncRNAs (*p* < 0.05). Later, LASSO regression was conducted to select and verify mRNAs or lncRNAs by the “glmnet” function of the R package. Last, this study established an mRNA- or lncRNA-based prognostic risk score according to summation of products of RNA expression multiplied by regression model (β), which was determined by the following formula: Risk_score = βlncRNA1 × lncRNA1 level + βlncRNA2 × lncRNA2 level + ⋅⋅⋅⋅⋅ + βlncRNAn × lncRNAn level or βmRNA1 × mRNA1 level + βmRNA2 × mRNA2 level + ⋅⋅⋅⋅⋅ + βmRNAn × mRNA level.

### Confirmation of Long Non-coding RNAs or mRNA Signature

This study assigned the enrolled cases and the survival data based on risk score. In addition, all cases were classified into high- or low-risk group according to the median risk score. Thereafter, this study drew the Kaplan–Meier (KM) survival curves for estimating high or low risk of the enrolled cases. Later, this study carried out a univariate analysis using the Cox proportional hazards regression model. Afterward, the risk score was utilized to compare the sensitivity and specificity of survival prediction, whereas time-dependent receiver operating characteristic (t-ROC) curves were drawn for evaluating 5-year prognosis prediction accuracy. Additionally, multivariate analysis was carried out to examine the independent prediction performance of mRNA or lncRNA risk score compared with additional clinical features. The conditional inference tree was constructed by “party” “tree” function of R package to further illustrate our results. *p* < 0.05 (two-sided) indicated statistical significance. The R software was employed for all analyses.

### Patient Specimens and Cell Culture

Altogether, 55 PCa samples and 55 matched non-carcinoma prostate samples were obtained from PCa cases treated at Affiliated Hospital of Jiangnan University fromMay 2013 to June 2018 (Table 1). All resected specimens were frozen within liquid nitrogen at once and stored under −80°C. Each subject provided written informed consent. The Research Ethics Committee of the Affiliated Hospital of Jiangnan University approved our study protocols carried out in line with the Declaration of Helsinki. Based on the median SLC17A9, LINC01679, or miR-3150a-3p expression, this study divided 55 PCa samples into a high- or a low-expression group. The human normal prostate epithelial RWPE-2 cells and human PCa cell lines (DU145, PC-3, LNCaP, C4-2B, and 22RV1) were provided by the American Type Culture Collection (ATCC, Rockville, MD, United States). All cell lines were cultivated within DMEM containing 10% fetal bovine serum (FBS, HyClone, Logan, UT, United States) as well as 1% penicillin-streptomycin (HyClone) under 37°C and 5% CO_2_ conditions.

### Bioinformatics Analysis

TargetScan database^3^ and starBase V2.0^4^ were applied in predicting miRNA target genes and lncRNA target miRNAs. Gene expression profiling interactive analysis (GEPIA) database^5^ was used to determine SLC17A9 expression in PCa and the correlation of LINC01679 and SLC17A9. Oncomine^6^ and UALCAN^7^ databases were used to determine SLC17A9 expression in PCa.

### Competitive Endogenous RNA Regulatory Network Establishment

Firstly, associations between those screened DElncRNAs and miRNAs and between those selected miRNAs and the selected differentially expressed genes (DEGs) were predicted by the starBase database. Thereafter, we adopted Cytoscape 3.5.1 software^8^ to visually map results.

### Cell Transfection

GenePharma was responsible for constructing vectors expressing LINC01679 [LINC01679-overexpression (OE); Shanghai, China] and vectors expressing SLC17A9 (SLC17A9-OE). Besides, we obtained empty vectors from GenePharma. The SLC17A9 or LINC01679-targeting small interfering RNA (siRNA) [SLC17A9- or LINC01679-knockdown (KD)], together with the corresponding negative control (NC), was provided by GenePharma (Shanghai, China). In the meantime, miR-3150a-3p inhibitor and miR-3150a-3p mimic, along with scrambled NC miRNA (NC inhibitor/mimic), were provided by Sigma-Aldrich. Lipofectamine 2000 Reagent (Invitrogen, Carlsbad, CA, United States) was utilized in cell transfection strictly following specific protocols. After reaching 30–50% confluency, we utilized 40 nM miRNAs, 20 nM siRNAs, and 15 nM vectors to transfect cells, separately, for 48 h by the use of Lipofectamine 2000 (Life Technologies) following specific instructions.

### Cell Counting Kit-8 Assay

Cells (4,000 cells/well) were inoculated into each well of the 96-well plates that contained 100 μl of culture medium. There were six wells set for each replicate. After 72 h of culture within the medium that contained 5% FBS, we inoculated cells within the 37°C incubator under 5% CO_2_ atmosphere overnight. Later, each well was added with 10 μl of CCK-8 solution (5 mg/ml, Beyotime Institute of Biotechnology, Shanghai, China) at 0, 12, 24, and 48 h, respectively, for CCK-8 assay, followed by another 1 h of culture. For blank control group, CCK-8 solution and culture medium were added into each well. Thereafter, the microplate reader (Bio-Tek, VT, United States) was adopted to measure absorbance (OD) value at 450 nm (OD 450) compared with control.

### Nuclear – Cytoplasmic Fractionation

This study utilized the NE-PER Cytoplasmic and Nuclear Extraction Reagents (Thermo Fisher Scientific) to separate the cytoplasmic fraction from the nuclear counterpart in line with specific protocols. Thereafter, total RNA was extracted by adopting the RNA Isolation Kit (Tiangen Biotech, Beijing, China). Afterward, the cytoplasmic-to-nuclear ratio of specific RNA expression was measured through qRT-PCR, with GAPDH being the cytoplasmic reference and U6 being the nuclear reference.

### Colony Formation Assay

After incubation in six-well plates at 500/well, the formation of cell colonies was observed after 2 weeks. Then, colonies were subjected to methanol fixation as well as 0.1% crystal violet staining under ambient temperature. Finally, colonies that contained at least 50 cells were regarded as the positive colonies under the microscope.

### Flow Cytometry

This study adopted the Annexin V-fluorescein isothiocyanate and propidium iodide (FITC/PI) kit (Beyotime Institute of Biotechnology, Shanghai, China) for detecting cell apoptosis through flow cytometric analysis in accordance with specific protocols.

### Transwell Assay

After resuspending in serum-free medium, cells from diverse groups were digested with pancreatin. Afterward, 200 μl of the cell suspension that contained altogether 5 × 10^3^ cells was added into the top Transwell chamber, whereas 500 μl of DMEM that contained 10% FBS was added into the bottom chamber for 24 h of incubation within a humidified incubator at 37°C. Subsequently, 4% formaldehyde was utilized to fix cells invading the Transwell chamber, and 0.1% crystal violet staining (Beyotime) was used to stain cells. Last, a microscope was utilized to detect cell invasion.

### Scratch Assay

Cells (5 × 10^5^/well) were inoculated into six-well plates. Later, we used the 200-μl pipette tip to create a wound on the confluent cell monolayer. Later, the inverted microscope was utilized to take images of wound closure at 0 and 24 h. Then, wound healing distance was examined.

### Tumor Xenograft Model

The Animal Care Committee of Fudan University Shanghai Cancer Center (Shanghai, China) approved our study protocols. Treated cells were injected subcutaneously in the 20 male BALB nude mice (4 weeks old; *n* = 5) *via* the right flank. Body weight (BW, g) and tumor volume were determined at intervals of 3 days. Each animal was sacrificed after 4 weeks to collect tumor tissues, images were taken, and tumor volume was measured. To be specific, tumor volume was measured by the following formula: Volume (mm^3^) = [width^2^ (mm^2^) × length (mm)]/2. The dissected tumor tissue was frozen within liquid nitrogen, followed by preservation under −80°C or 10% formalin fixation, paraffin embedding, sectioning, and staining.

### Dual-Luciferase Reporter Assay

A luciferase construct containing wild-type (WT) and mutated (MUT) binding site vectors of LINC01679 3′ -untranslated region (3′UTR) or WT and MUT binding site vectors of SLC17A9 3′UTR (Promega, Madison, Wisconsin, United States) was co-transfected with scramble or miR-3150a-3p mimic/inhibitor into cells grown within 24-well plates by the use of Lipofectamine 3000 (Thermo Fisher Scientific, Inc). Later, we used a dual-luciferase assay system (Promega) to analyze luciferase activities of transfected cells in accordance with specific instructions.

A luciferase construct containing WT and MUT binding site vectors of SLC17A9 3′UTR (Promega, Madison, Wisconsin, United States) was co-transfected with NC/vector or LINC01679-KD/LINC01679-OE into cells grown within 24-well plates by the use of Lipofectamine 3000 (Thermo Fisher Scientific, Inc). Later, we used a dual-luciferase assay system (Promega) to analyze luciferase activities of transfected cells in accordance with specific instructions.

### RNA Pull-Down Assay

Probe-ATB or probe-control was transcribed from ATB shRNA lentivector and labeled *in vitro* by Biotin RNA Labeling Mix (Roche, Basel, Switzerland) to carry out RNA pull-down assay. Secondary structure was formed through biotin-labeled RNAs by adopting the RNA structure buffer (Thermo Fisher Scientific, MA, United States). Then, RNA immunoprecipitation wash buffer (500 μl, Thermo Fisher Scientific) was utilized to rinse Streptavidin beads (Thermo Fisher Scientific) thrice, while the beads were later mixed with biotinylated RNAs overnight under 4°C. Later, the magnetic field was applied to separate the overnight mixture to obtain the streptavidin bead–RNA complexes. Afterward, complexes were mixed with cell lysates before 1 h of incubation on the rotator under ambient temperature. Again, the magnetic field was applied to separate the mixture after incubation for obtaining streptavidin bead–RNA–protein complexes.

### qRT-PCR

Quick-RNA™ Microprep Kit (Zymo, CA, United States) was employed to extract the total cellular RNA. Meanwhile, the PrimeScript™ RT Reagent Kit equipped with gDNA Eraser (TaKaRa, Shiga, Japan) was adopted to prepare cDNA in accordance with the manufacturer’s instructions. Then, the TB Green Premix ExTaq II (Tli RNaseH Plus, TaKaRa) was applied for qRT-PCR, whereas the LightCycler^®^ 96 Instrument (Roche, Switzerland) was employed for analysis. Then, we determined fold changes (FC) by the ΔΔCt approach, with U6 or GAPDH being the endogenous control. Table 2 lists the primers used.

### Western Blotting Analysis

After washing with PBS, cells were lysed with the pre-chilled lysis buffer. Later, the collected cell lysates were subjected to 15 min of centrifugation at 14,000 × *g* and 4°C and boiling with 5 × sample buffer (BSA; Thermo Fisher Scientific, Inc., CA, United States) after the protein content was measured. Afterward, WB assay was performed on those protein samples. To carry out WB, the 4%–20% precasting gel (Bio-Rad Laboratories) was used to transfer protein onto the nitrocellulose membranes, and then 5% skim milk was utilized to block the membranes (Bio-Rad Laboratories) for 1 h under ambient temperature. Later, specific antibodies (dilution, 1:1,000), including anti-SLC17A9, anti-VEGF, anti-Bax, anti-Ki67, anti-MTA1, anti-Bcl-2, anti-MMP-2, and GAPDH (Santa Cruz, CA, United States), were used to block membranes. Subsequently, secondary antibodies (Santa Cruz) were utilized to incubate membranes at ambient temperature for 1 h, followed by visualization using the ECL solution (Bio-Rad Laboratories). Finally, images were taken using the chemiluminescence imaging system (Mini HD9; UVitec, Cambridge, United Kingdom).

### Statistical Analysis

The relative change in LINC01679 expression > 0.8 (median value) was deemed as high expression. Correlations between LINC01679 level and clinicopathological factors were examined by chi-square test. Besides, a KM curve was drawn to analyze patient survival by log-rank test. Results were displayed as means ± standard deviation (SD). SPSS22.0 was employed to carry out statistical analysis. Data across diverse groups were compared through ANOVA and least significant difference (LSD) test, whereas those of two groups were examined through Student’s *t*-test. A difference of *p* < 0.05 indicated statistical significance. Each experiment was conducted thrice.

## Results

### Identification of Significant Differentially Expressed Long Non-coding RNAs for Prostate Cancer Prognosis

**FIGURE 1:**
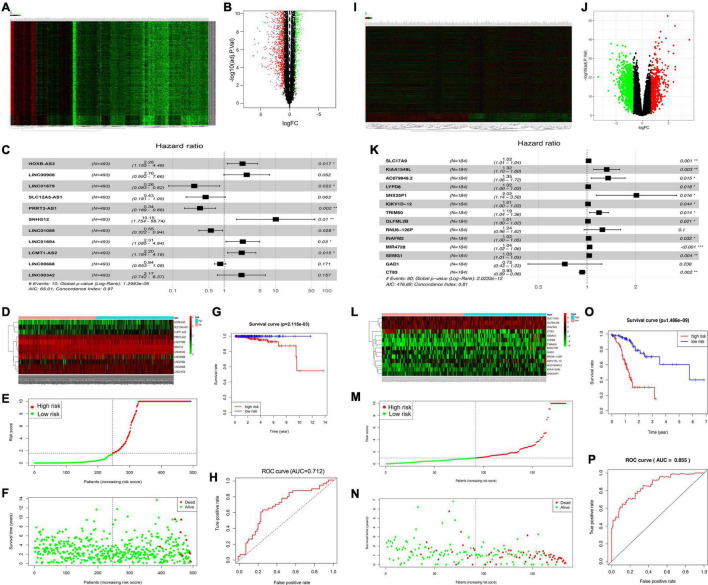
Identification of significant PCa-related DElncRNAs and DEGs. Heat map demonstrating the DElncRNAs **(A)** and DEGs **(I)**. Volcano plot showing the DElncRNAs **(B)** and DEGs **(J)**, FC, fold change. **(C)** Forest map showing HRs (95% CIs) together with *p*-values for those screened DElncRNAs, and DEGs **(K)** by univariate Cox proportional hazards analysis. Heat map displaying the expression levels of OS-related DElncRNAs **(D)** and DEGs **(L)** in the high-risk and low-risk groups. **(E,M)** Risk curve plotted according to risk scores of all samples. **(F,N)** Scatterplot drawn according to patient survival status, where red and green dots denote death and survival, respectively. **(G,O)** KM curve showing the survival between low- and high-risk groups according to median risk score and risk model. **(H,P)** AUC values of clinical characteristics and risk score based on ROC curves.

This study discovered DElncRNAs between cancer and non-carcinoma samples. Altogether, 2,974 DElncRNAs (namely, 1,598 with downregulation and 1,376 with upregulation) were discovered by the “edgeR” function of the R package. Thereafter, the identified DElncRNAs were incorporated in the construction of a volcano plot (Figure 1A) as well as a heat map showing the 200 most significant genes (Figure 1B). Among them, 11 PCa-related DElncRNAs were found to be markedly related to OS of TCGA-derived PCa cases (*p* < 0.01) through Cox proportional hazards analysis, which included five low-risk lncRNAs [LINC01679, SLC12A5-AS1, PRRT3-AS1, LINC01088, and LINC00668; hazard ratio (HR) < 1] and six high-risk ones (HOXB-AS3, LINC00908, SNHG12, LINC01694, LCMT1-SA2, and LINC00342; HR > 1) (Figure 1C). The above-identified 11 DElncRNAs were subsequently adopted to construct the best prognosis prediction nomogram for PCa-associated lncRNAs (Figure 1D). Risk score together with relevant survival of PCa cases was illustrated by risk curve and scatterplot. As a result, the greater risk score indicated the higher risk of mortality (Figures 1E,F). As revealed by KM survival analysis, high-risk cases showed reduced OS relative to low-risk patients (*p* = 2.115e-03; Figure 1G). Besides, time-dependent receiver operating characteristic (ROC) curve analysis showed that the area under the ROC (AUC) value for the survival-related lncRNA prognosis signature was 0.712 (Figure 1H).

### Identification of Significant Prostate Cancer-Related Differentially Expressed Genes

This study obtained significant DEGs between cancer and non-carcinoma samples. Altogether, 1,938 DEGs (namely, 1,488 with downregulation and 450 with upregulation) were discovered by the “edgeR” function of the R package. The above DEGs were later incorporated into the construction of a volcano plot for DEGs (Figure 1I) together with a heat map for the 200 most significant genes (Figure 1J). Of these genes, 14 DEGs were found to be markedly related to OS of TCGA-derived PCa cases (*p* < 0.01) through Cox proportional hazards analysis, which included 2 low-risk DEGs (GAD1 and CT83; HR < 1) and 12 high-risk ones (SLC17A9, KIAA1549L, AC079949.2, LYPD8, SNX25P1, IGKV1D-12, TRIM50, OLFML2B, SEMG1, RNU6-126P, INAFM2, and MIR4728; HR > 1) (Figure 1K). These 14 DEGs were later included to construct the best prognosis prediction nomogram for DEGs related to PCa prognosis (Figure 1L). A greater risk score stood for the greater risk of mortality (Figures 1M,N). According to KM survival analysis, high-risk cases showed reduced OS relative to low-risk cases (*p* = 1.486e-09; Figure 1O). Besides, time-dependent ROC curve analysis showed that the AUC value for the survival-related mRNA prognosis signature was 0.855 (Figure 1P).

### Competitive Endogenous RNA Regulatory Network Construction in Prostate Cancer

**FIGURE 2:**
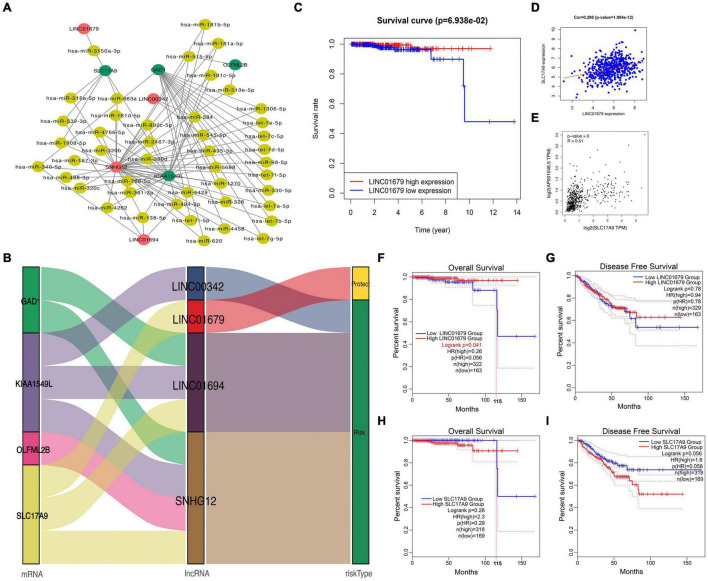
Construction of a ceRNA regulatory network for PCa. **(A,B)** Construction of the four lncRNAs–mRNAs co-expression networks for predicting the OS of PCa, and network visualization by Sankey diagram and Cytoscape. **(C)** KM analysis on high and low LINC01679 levels. **(D)** Correlation analysis between LINC01679 expression and SLC17A9 expression based on TCGA database **(D)** and GEPIA database **(E)**. GEPIA database was used to analyze the role of LINC01679 in OS **(F)** and DFS **(G)** of PCa patients and the role of SLC17A9 in OS **(H)** and DFS **(I)** of PCa patients.

To further understand the role of DElncRNAs within PCa, the present work constructed the ceRNA regulatory network according to lncRNAs–miRNAs–mRNAs for PCa. Firstly, 14 DEGs and 11 DElncRNAs acquired in the previous section were utilized; then, 70,727 pairs of overlapped miRNAs and lncRNAs and 2,012,127 pairs of overlapped mRNAs and miRNAs were identified based on the starBase database. According to our findings, four mRNAs were chosen to construct the ceRNA network. Last, the ceRNA network for PCa was established based on 4 DElncRNAs, 45 DEmiRNAs, and 4 DEmRNAs (Figure 2A). As observed from Figure 2B, LINC01679 (lncRNA)–SLC17A9 (mRNA) was the only pair of lncRNA–mRNA in the protection group. Furthermore, KM analysis revealed that cases showing low expression of LINC01679 had reduced OS relative to those with high expression (Figure 2C). LINC01679 was co-expressed with SLC17A9 in PCa, and LINC01679 expression was positively associated with SLC17A9 expression (Figure 2D). The GEPIA website pooled the data from TCGA and Genotype-Tissue Expression (GTEx) datasets. The GTEx dataset can complement the lack of control data in the TCGA dataset. The inclusion of GTEx data will make the results of the analysis more accurate. The results are shown in Figure 2E, which similarly suggested that LINC01679 expression was positively associated with SLC17A9 expression. In addition, we further analyze the role of LINC01679 and SLC17A9 in the OS and disease-free survival (DFS) of PCa based on the GEPIA database. The results showed that LINC01679 expression was significantly associated with OS of PCa (Figure 2F) and has not been significant to the DFS of PCa (Figure 2G). However, it was obvious from Figure 2G that the rate of the DFS for PCa patients with high LINC01679 expression was superior to that of PCa patients with low LINC01679 expression. In addition, SLC17A9 expression is not significant to the OS (Figure 2H) and the DFS of PCa (Figure 2I). When survival time exceeds 115 months, the survival rate of PCa patients with high SLC17A9 expression is significantly higher than that of PCa patients with low SLC17A9 expression. Next, LINC01679 expression was significantly related to PSA and gleason grade (Table 1).

**TABLE 1:** The Relationship between LINC01679 and clinicopathological characteristics in 55 patients with prostate cancer.

Parameters	No. of cases	LINC01679 expression		*p*-values
		Low	High	
Age (years)				0.218
≥60	28	15	13	
<60	27	10	17	
Differentiation				0.96
Poor	31	14	17	
Well/moderate	24	11	13	
PSA				0.004*
≥23.6	28	18	10	
<23.6	27	7	20	
Gleason score				0.002*
≥7	29	19	10	
<7	26	6	20	

PSA, prostate-specific antigen; *p < 0.05.

**TABLE 2:** The primer sequence of qRT-PCR.

Gene	Sequence (5′-3′)
LINC01679	F: TGCCACTCGTGAGAACTGTCTA R: GATAGGCTCTGCAAGACACC
miR-3150a-3p	F: CCAGAGGGTCCACTCCAGTTTTCCAG R: GACTCAAGGGTGTCGTCT
SLC17A9	F: TGGGTTGTGGGGTCATGGG R: TACTTCTCTGTGGCATGATGGCT
GAPDH	F: GAACGAGCCGAGTGAAGCC R: CTTTGACTGCTTTCCCACCGG
U6	F: GCAGTGGACGACGAGTAGGA R: GCACACGAAGCAGGAAGCTA

### LINC01679 was Correlated With Clinicopathologic Characteristics and Regulated Cell Proliferation, Invasion, Migration, Apoptosis, Tumorigenesis *in vitro and vivo*

**FIGURE 3:**
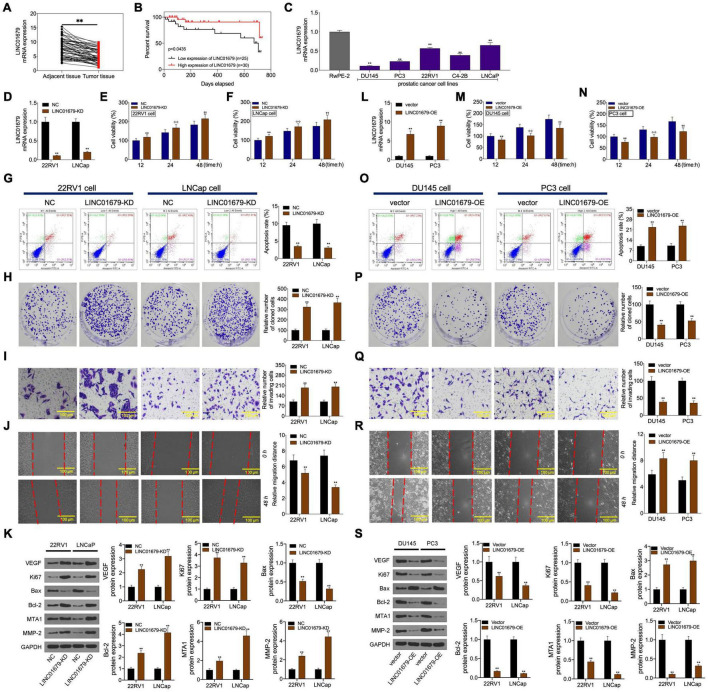
LINC01679 regulated cell proliferation, apoptosis, migration, and invasion *in vitro.* **(A)** qRT-PCR assay was conducted to detect LINC01679 expression in PCa patients. **(B)** KM survival analysis on high and low LINC01679 expression. **(C)** LINC01679 levels within PCa cells measured by qRT-PCR assay. LINC01679 expression **(D,L)**, cell viability **(E,F,M,N)**, cell apoptosis **(G,O)**, proliferation **(H,P)**, invasion **(I,Q)**, migration **(J,R)**, and protein expression of VEGF, Ki67, Bax, Bcl-2, MTA1, and MMP-2 **(K,S)** of cells transfected with siRNA-LINC01679 or LINC01679-expressing vector determined by qRT-PCR, CCK-8, flow cytometry, colony formation, Transwell, scratch, and WB assays. The experiment was repeated thrice independently; ** vs. NC or vector group, *p* < 0.01; ✣✣ vs. vector at 24 h group; †† vs. vector at 48 h group.

Firstly, our results showed that low expression of LINC01679 was significantly correlated with high PSA level (≥23.6 ng/ml) and high Gleason score (≥7) (P < 0.01, Table 1). Then LINC01679 levels within cancer samples markedly reduced relative to matched non-carcinoma samples (Figure 3A). Patients in the low LINC01679 expression group displayed poor survival (Figure 3B). In addition, LINC01679 expression within PCa cells decreased significantly, relative to normal control cells (Figure 3C). After siRNA targeting LINC01679 was transfected into 22RV1 and LNCap cells, LINC01679 expression in the LINC01679-KD group decreased significantly (Figure 3D). Moreover, according to flow cytometric, CCK-8, colony formation, scratch, and Transwell assays, LINC01679-KD enhanced cell growth, invasion, and migration, but suppressed their apoptosis *in vitro* (Figures 3E–J). Furthermore, LINC01679-KD upregulated the expression of VEGF, Ki67, Bcl-2, MTA1, and MMP2, while downregulating that of Bax at the protein level (Figure 3K). Meanwhile, LINC01679-expressing vector was transfected into DU145 and PC3 cells with low LINC01679 expression, so as to upregulate the LINC01679 levels (Figure 3L). As a result, cell viability was inhibited, cell apoptosis increased, and invasion and migration were attenuated (Figures 3M–R). Moreover, LINC01679-OE reduced VEGF, Ki67, Bcl-2, MTA1, and MMP2 levels, while increasing Bax expression at the protein level (Figure 3S).

**FIGURE 4:**
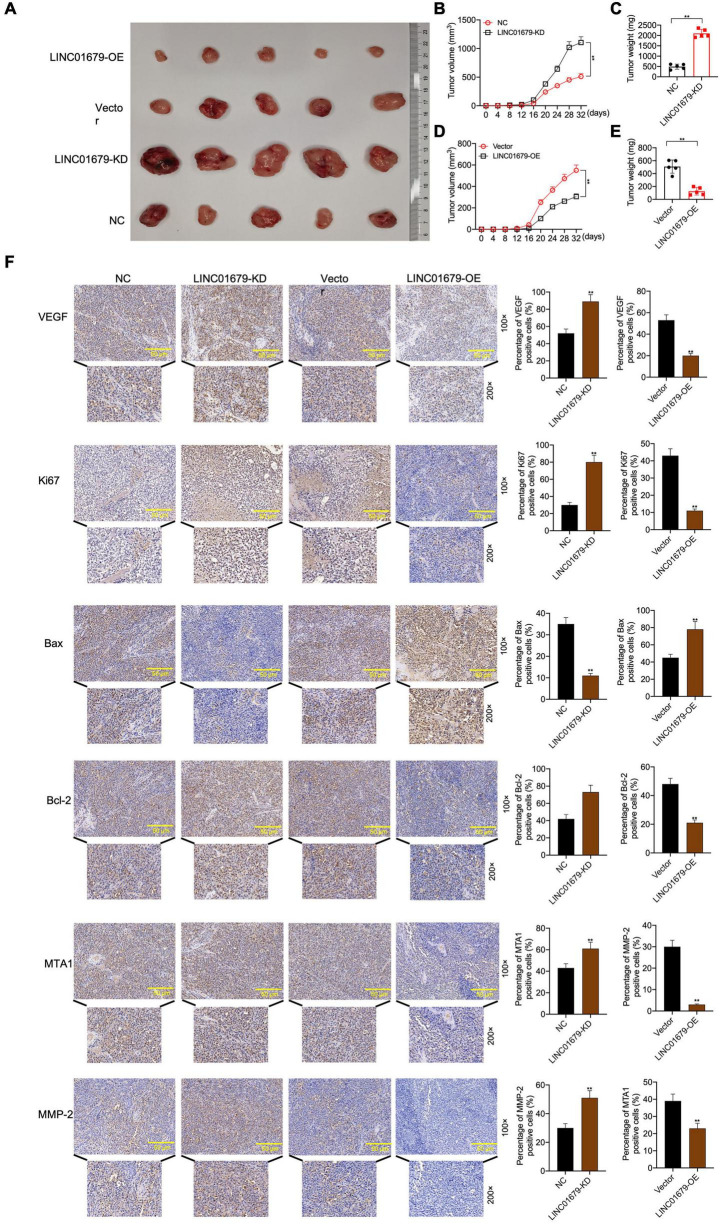
LINC01679 regulated tumorigenesis *in vivo.* **(A)** The nude mouse tumor xenograft assay displaying the oncogenicity of LINC01679 inhibition or overexpression (*n* = 5). Quantitative analysis on tumor growth **(B)** and tumor weight **(C)** after siRNA-LINC01679 treatment. Quantitative analysis on tumor growth **(D)** and tumor weight **(E)** after LINC01679-expressing vector treatment. **(F)** Immunohistochemical staining of VEGF, Ki67, Bax, Bcl-2, MTA1, and MMP-2 in tumor tissues. The experiment was repeated thrice independently; ** vs. NC or vector group, *p* < 0.01.

The nude mouse tumor xenograft experiment showed that LINC01679-KD transfected 22RV1 cells had increased oncogenicity compared with untreated 22RV1 cells, whereas LINC01679-OE transfected PC3 cells had decreased oncogenicity relative to untreated PC3 cells (Figures 4A–E). VEGF, Ki67, Bax, Bcl-2, MTA1, and MMP2 expression in cancer tissues was consistent with those in cancer cells, as revealed by immunohistochemical staining (Figure 4F).

### LINC01679 Played a Role of Sponging miR-3150a-3p in Prostate Cancer

**FIGURE 5:**
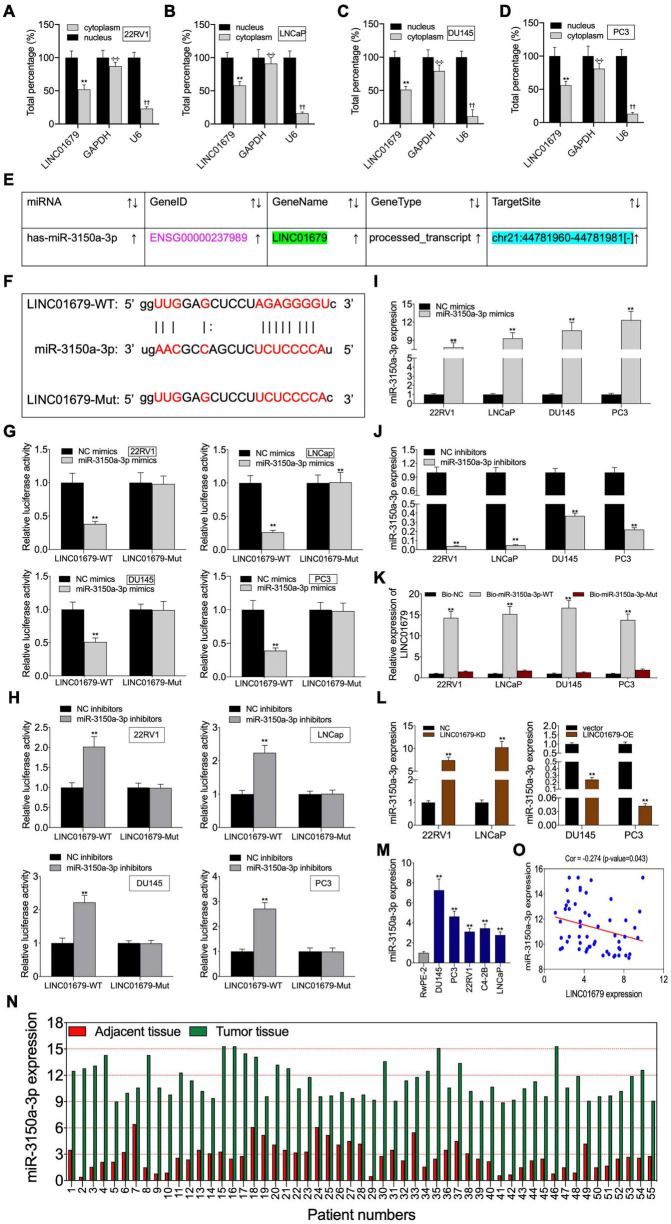
LINC01679 acted as a sponge of miR-3150a-3p in PCa. **(A–D)** Nuclear–cytoplasmic fractionation was performed to investigate LINC01679 expression in the cytoplasm and nucleus of PCa cells. **(E)** miR-3150a-3p was predicted as a potential target of LINC01679 by the starBase website. **(F)** Specific binding regions between LINC01679 sequence and miR-3150a-3p sequence analyzed by the online analysis software. **(G,H)** Binding of LINC01679 to miR-3150a-3p verified by dual-luciferase reporter gene assay. **(I,J)** miR-3150a-3p expression in PCa cell lines after miR-3150a-3p mimics/or inhibitors treatment determined by qRT-PCR. **(K)** Dramatic enrichment of LINC01679 was detected in biotinylated miR-3150a-3p by RNA pull-down assay. **(L)** LINC01679 expression after siRNA-LINC01679 or LINC01679-expressing vector treatment determined by qRT-PCR. **(M,N)** LINC01679 expression in PCa cell lines and patients measured by qRT-PCR. **(O)** Spearman correlation analysis between LINC01679 expression and miR-3150a-3p expression. The experiment was repeated thrice independently; ** vs. NC or vector group, *p* < 0.01; ✣✣ vs. nucleus (GAPDH) group; †† vs. nucleus (U6) group.

To investigate the function of LINC01679 to sponge miRNA, we separated the nuclear fraction from the cytoplasmic counterpart. As a result, cytoplasmic localization of LINC01679 was mainly found (Figures 5A–D). Based on the starBase website, we predicted miR-3150a-3p as the candidate LINC01679 target (Figure 5E). Then, based on bioinformatics analysis, LINC01679 had a binding site for miR-3150a-3p (Figure 5F). To validate the direct interaction of LINC01679 with miR-3150a-3p, this study carried out a luciferase reporter assay. As observed, miR-3150a-3p mimics remarkably decreased the LINC01679-WT reporter luciferase activity, but the difference was not significant compared with the LINC01679-Mut reporter (Figure 5G); by contrast, miR-3150a-3p inhibitors dramatically elevated LINC01679-WT reporter luciferase activity, and the difference was not significant compared with the LINC01679-Mut reporter (Figure 5H). In addition, miR-3150a-3p mimics and miR-3150-3p inhibitors were transfected into PCa cell lines, respectively, to increase or decrease miR-3150a-3p levels (Figures 5I,J). LINC01679 was dramatically enriched into the biotinylated miR-3150a-3p, as confirmed by RNA pull-down assay (Figure 5K). Moreover, LINC01679-KD increased miR-3150a-3p expression in 22RV1 and LNCap cells, whereas LINC01679-OE inhibited miR-3150a-3p expression in DU145 and PC3 cells (Figure 5L). Next, high miR-3150a-3p levels were measured within PCa cell lines and tissues (Figures 5M,N), which showed negative correlation with LINC01679 level among PCa cases (Figure 5O).

### miR-3150a-3p Regulated Cell Proliferation, Invasion, Migration, and Apoptosis *in vitro*

**FIGURE 6:**
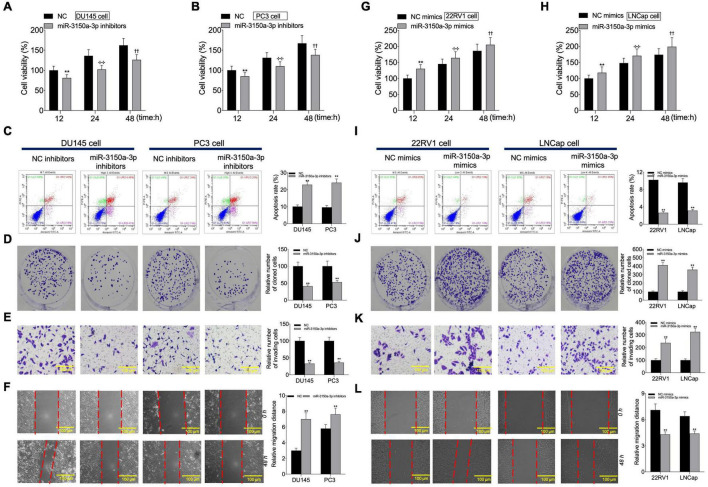
miR-3150a-3p regulated cell proliferation, apoptosis, migration, and invasion *in vitro.* Cell viability **(A,B,G,H)**, cell apoptosis **(C,I)**, proliferation **(D,J)**, invasion **(E,K)**, and migration **(F,L)** of cells transfected with miR-3150a-3p inhibitors or miR-3150a-3p mimics determined by CCK-8, flow cytometry, colony formation, Transwell, and scratch assays. The experiment was repeated thrice independently; ** vs. NC inhibitors or NC mimics group, *p* < 0.01; ✣✣ vs. vector at 24 h group; †† vs. vector at 48 h group.

According to CCK-8, flow cytometric, colony formation, scratch, and Transwell assays, miR-3150a-3p inhibitors inhibited PC3 and DU145 cell growth, migration, and invasion, and enhanced their apoptosis (Figures 6A–F). Besides, miR-3150a-3p mimics enhanced 22RV1 and LNCap cell growth, invasion, and migration, and suppressed their apoptosis (Figures 6G–L).

### SLC17A9 Acted as an miR-3150a-3p Target Gene and Modulated Cell Growth, Invasion, Migration, and Apoptosis *in vitro*

**FIGURE 7:**
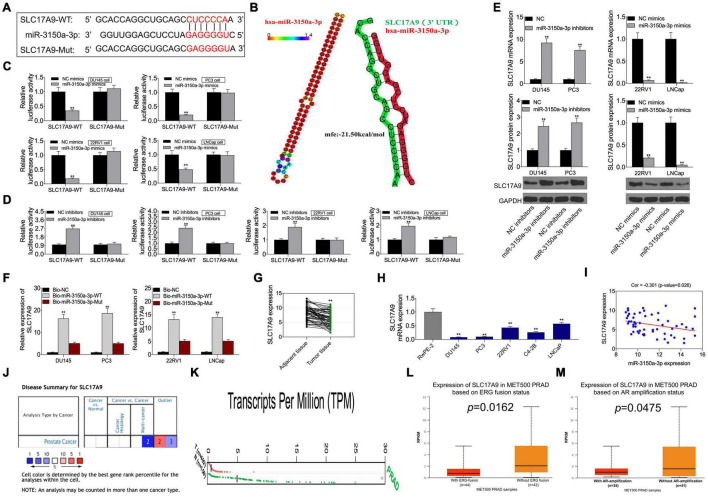
**(A)** SLC17A9 acted as a target gene of miR-3150a-3p in PCa. SLC17A9 was predicted as a potential target of miR-3150a-3p by the starBase website. **(B)** The stem-loop structure of hsa-miR-3150a-3p was displayed using the RNAhybrid program, and the SLC17A9 target binding sites on the whole miR-3150a-3p sequences were predicted. **(C,D)** Binding of SLC17A9 to miR-3150a-3p verified by dual-luciferase reporter gene assay. **(E)** miR-3150a-3p expression in PCa cell lines transfected with miR-3150a-3p mimics or inhibitors determined by qRT-PCR and WB assays. **(F)** Dramatic enrichment of SLC17A9 was detected in biotinylated miR-3150a-3p by RNA pull-down assay. **(G,H)** SLC17A9 expression in PCa cell lines and patients measured by qRT-PCR. **(I)** The correlation analysis between SLC17A9 expression and miR-3150a-3p expression. SLC17A9 expression analysis in PCa samples based on Oncomine database **(J)**, GEPIA database **(K)**, and UALCAN database **(L,M)**. ** vs. NC mimics/inhibitors/Bio-NC/RwPE-2 group.

This study predicted SLC17A9 as the candidate miR-3150a-3p target based on the starBase website, and it was confirmed that miR-3150a-3p contained the binding site for SLC17A9 (Figure 7A). Subsequently, hsa-miR-3150a-3p’s stem-loop structure was revealed by the RNAhybrid program^9^ and visualized using ‘‘RNAcofold’’ and ‘‘RNAfold’’ packages^10^. Then, the binding sites of SLC17A9 target on the whole miR-3150a-3p sequences were predicted (Figure 7B). Luciferase reporter assay confirmed that SLC17A9 directly interacted with miR-3150a-3p (Figures 7C,D). Moreover, miR-3150a-3p inhibitors increased SLC17A9 level within DU145 and PC3 cells, but miR-3150a-3p mimics inhibited that in 22RV1 and LNCap cells (Figure 7E). SLC17A9 was dramatically enriched into the biotinylated miR-3150a-3p, as revealed by the RNA pull-down assay (Figure 7F). In addition, SLC17A9 was significantly downregulated in PCa cells and tumor tissues (Figures 7G,H). SLC17A9 level showed significant negative correlation with the miR-3150a-3p level among PCa cases (Figure 7I). We wonder whether the expression level of SLC17A9 in castration-resistant prostate cancer (CRPC) tissues or metastatic tissues could be analyzed by using three public databases, including Oncomine, GEPIA, and UALCAN. However, the results of the Oncomine database do not have the data of SLC17A9 expression in PCa (Figure 7J). Then, the GEPIA database showed that SLC17A9 expression in tumor samples was significantly lower than that in normal samples (Figure 7K). SLC17A9 expression in metastatic PCa was decreased manifesting downregulated SLC17A9 expression in PCa patients with ERG fusion status and AR amplification status (Figures 7L,M).

**FIGURE 8:**
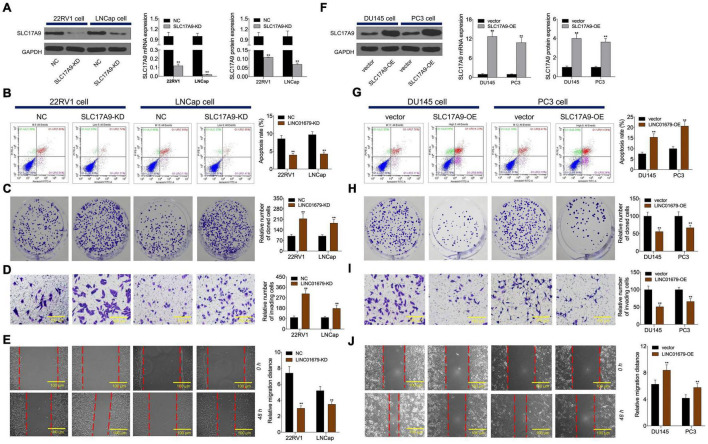
SLC17A9 regulated cell proliferation, apoptosis, migration, and invasion *in vitro*. SLC17A9 expression **(A,F)**, cell apoptosis **(B,G)**, proliferation **(C,H)**, invasion **(D,I)**, and migration **(E,J)** of cells transfected with siRNA-SLC17A9 or SLC17A9-expressing vector determined by qRT-PCR, WB, flow cytometry, colony formation, Transwell, and scratch assays. The experiment was repeated thrice independently; ** vs. NC or vector group, *p* < 0.01.

To investigate whether SLC17A9 affected PCa cell growth, invasion, migration, and apoptosis, SLC17A9-KD and SLC17A9-OE were transfected into PCa cells to downregulate (Figure 8A) or upregulate (Figure 8F) their expression. The results showed that SLC17A9-KD inhibited cell apoptosis and promoted invasion and migration (Figures 8B–E), whereas SLC17A9-OE had opposite effects (Figures 8G–J).

### miR-3150a-3p Regulated Cell Growth, Invasion, Migration, and Apoptosis by Targeting SLC17A9 *in vitro*

**FIGURE 9:**
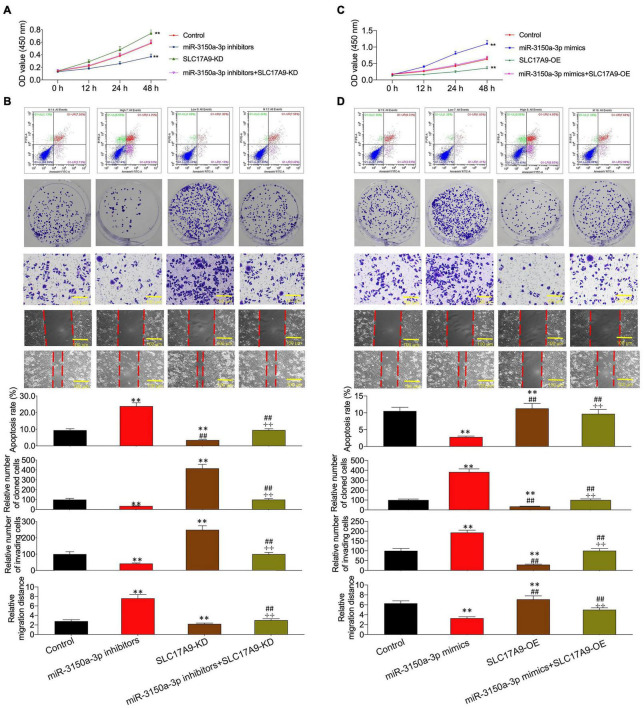
miR-3150a-3p regulated cell proliferation, apoptosis, migration, and invasion *via* targeting SLC17A9 *in vitro.* Cell viability **(A,C)**, cell apoptosis, proliferation, invasion, and migration **(B,D)** of cells co-transfected with miR-3150a-3p inhibitors and siRNA-SLC17A9 or miR-3150a-3p mimics and SLC17A9-expressing vector determined by CCK-8, flow cytometry, colony formation, Transwell, and scratch assays. Each independent assay was conducted in triplicate; ** vs. control group, *p* < 0.01.

Functions of miR-3150a-3p inhibitors in promoting cell apoptosis and inhibiting proliferation, migration, and invasion were suggested using SLC17A9-KD (Figures 9A,B). In contrast, miR-3150a-3p mimics’ role in inhibiting cell apoptosis and promoting proliferation, invasion, and migration was abolished by SLC17A9-OD (Figures 9C,D).

### SLC17A9 Was Involved in LINC01679-mediated Inhibition of Prostate Cancer Progression

**FIGURE 10:**
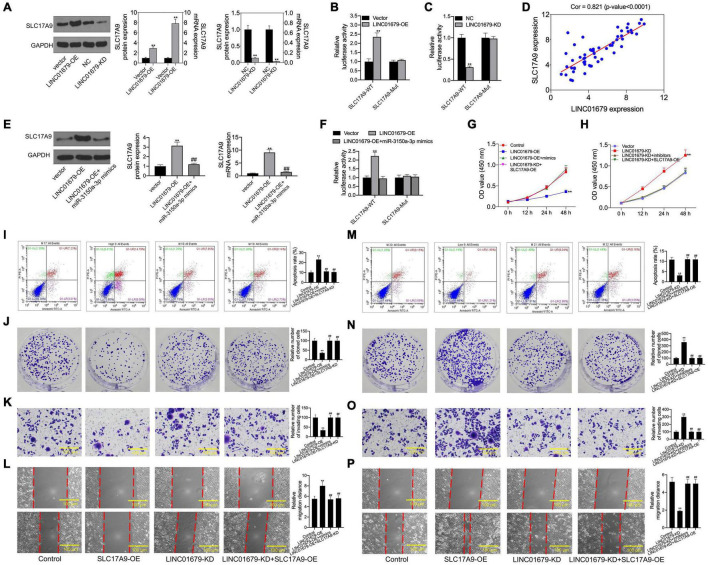
SLC17A9 involved in the LINC01679-mediated inhibition of PCa progression. **(A)** SLC17A9 expression in PCa cell lines transfected with LINC01679-siRNA or LINC01679-expressing vector measured through qRT-PCR and Western blotting. **(B,C)** Binding of SLC17A9 to LINC01679 verified by dual-luciferase reporter gene assay. **(D)** The correlation analysis between LINC01679 expression and SLC17A9 expression. **(E)** SLC17A9 expression in PCa cell lines co-transfected with LINC01679-expressing vector and miR-3150a-3p mimics by measured through qRT-PCR and Western blotting. **(F)** The interaction among SLC17A9, miR-3150a-3p, and LINC01679 verified by dual-luciferase reporter gene assay. Cell viability **(G,H)**, cell apoptosis **(I,M)**, proliferation **(J,N)**, invasion **(K,O)**, and migration **(L,P)** of cells after transfection with LINC01679-expressing vector and siRNA-SLC17A9 or LINC01679-expressing vector and SLC17A9-expressing vector determined by CCK-8, flow cytometry, colony formation, Transwell, and scratch assays. Each independent assay was conducted in triplicate; ** vs. control group, *p* < 0.01; ## vs. LINC01679-OE group.

Then, SLC17A9 protein and mRNA expression was under the regulation by LINC01679 (Figure 10A). To further determine the interaction of LINC01679 with SLC17A9, we carried out luciferase reporter assay. The results showed that transfection with LINC01679-OE markedly upregulated the SLC17A9-WT reporter luciferase activity, but still the difference was not significant compared with the SLC17A9-Mut reporter (Figure 10B), whereas transfection with LINC01679-KD markedly downregulated the SLC17A9-WT reporter luciferase activity (Figure 10C). SLC17A9 level showed a positive relationship with LINC01679 level within PCa cases (Figure 10D). Whether LINC01679 regulates SLC17A9 expression through targeting miR-3150a-3p needs to be further studied. The results of this study showed that SLC17A9 protein and mRNA expression saw a synchronous rise by transfection with LINC01679-OE, but this effect disappeared in cells co-transfected with LINC01679-OE and miR-3150a-3p mimics (Figure 10E). The effect of transfection with LINC01679-OE induced the SLC17A9-WT reporter luciferase activity, and the increase was reversed by miR-3150a-3p mimics (Figure 10F). According to the experimental results, upregulating miR-3150a-3p or downregulating SLC17A9 abolished the effect of LINC01679 overexpression on suppressing cell growth, invasion, and migration, and enhancing cell apoptosis (Figures 10G–P). However, the positive functions of LINC01679 knockdown in cell growth, migration, and invasion were attenuated in miR-3150a-3p inhibitor or SLC17A9-expressing vector transfected cells. Collectively, this study confirmed that LINC01679 was a ceRNA that inhibited PCa development through modulating the miR-3150a-3p/SLC17A9 axis.

## Discussion

Some lncRNA-based prognosis prediction nomograms are established for PCa (Alahari et al., 2016). As far as we know, few lncRNA signatures are established for PCa cases. The present work was conducted among PCa cases for predicting their survival based on the mRNA and lncRNA signatures. In the present work, OS-related DEGs or DElncRNAs were completely selected through univariate and bioinformatics analyses. Then, the significant mRNAs and lncRNAs were incorporated for the construction of the prognosis prediction model. Later, Cox regression, KM, and ROC curve analyses were performed to confirm the value of the constructed mRNA and lncRNA signatures in prognosis prediction. A ceRNA network of PCa was established by lncRNA signature, mRNA signature, and their common miRNAs. Interestingly, the LINC01679/miR-3150a-3p/SLC17A9 axis was the only axis in the protection group in the network involving 4 DElncRNAs, 45 DEmiRNAs, and 4 DEmRNAs. lncRNAs have been well recognized to sponge candidate miRNAs as the ceRNAs, so as to regulate cancer development. For instance, lncRNA FOXD2-AS1 is the ceRNA that modulates thyroid cancer by combining with miR-7-5p (Liu et al., 2019). In addition, LINC-PINT suppresses the genesis of non-small cell lung cancer (NSCLC) as the miR-218-5p sponge through MAFG-AS1, and modulates miR-339-5p expression to promote breast cancer (BC) aggressiveness (Zhang L. et al., 2019). LINC01679’s function in cancer together with the related mechanism has not been reported. Therefore, it was speculated that LINC01679 might function as the ceRNA in PCa. LINC01679 expression in PCa cells and tissues decreased significantly, compared with surrounding tissues or normal control cells, and the low LINC01679 expression group displayed a poor survival. Our results showed that LINC01679 overexpression inhibited cell proliferation, migration, invasion, and tumor growth, and induced cell apoptosis *in vitro* and *in vivo*. Therefore, LINC01679 might be an anti-oncogene in PCa. Therefore, the LINC01679/miR-3150a-3p/SLC17A9axis was used for follow-up research.

Many studies have suggested that miRNAs function as tumor suppressors or oncogenes to modulate cancer genesis and progression. For example, miR-193a-5p silencing enhances the chemosensitivity to docetaxel in PCa (Yang et al., 2017). miR-584-5p overexpression decreases gastric cancer (GC) cell growth and increases their apoptosis (Li et al., 2017). Nonetheless, as an important target of LINC01679, the role of miR-3150a-3p in tumor is still unclear.

Our results showed that miR-3150a-3p expression in PCa cells and tissues increased significantly, compared with matched surrounding tissues or normal control cells; besides, patients with high miR-3150a-3p expression displayed poor survival. According to our results, miR-3150a-3p mimics enhanced PCa cell growth, invasion, and migration, but inhibited their apoptosis. From the above, miR-3150a-3p was identified as an oncogene in PCa. Furthermore, miR-3150a-3p showed direct interaction with LINC01679. Rescue experiments proved that miR-3150a-3p upregulation abolished the suppression of LINC01679 overexpression on cell proliferation, invasion, and migration, and the promotion on cell apoptosis. Collectively, LINC01679 regulates PCa development by the sponge of miR-3150a-3p.

Solute carrier family 17 member 9 (SLC17A9) has been discovered as a vesicular nucleotide transporter (VNUT) in recent years, and it also belongs to the transmembrane protein family related to small-molecule transport (Wu et al., 2020). It has been reported that SLC17A9 upregulation is related to the dismal prognostic outcome of GC and colorectal cancer (CRC) (Li et al., 2019; Yang et al., 2019). However, this study indicated that SLC17A9 level was in direct proportion to LINC01679, and low expression of SLC17A9 predicted the poor prognosis of PCa. This showed that the same gene played different roles in different tumors. Interestingly, when survival time exceeds 115 months, the survival rate of PCa patients with high SLC17A9 expression and the rate of PCa patients with high LINC01679 expression are both significantly higher than that of PCa patients with low SLC17A9 expression and PCa patients with low LINC01679 expression. These also proved that the role of LINC01679 in OS of PCa patients was consistent with that of SLC17A9 in OS of PCa patients. The ERG gene in PCa is mainly regulated by the TMPRSS2-ERG fusion gene. It reported that nearly half of PCa cases express the TMPRSS2-ERC fusion gene (Wang et al., 2017). Therefore, the ERG gene should be an important marker of PCa, and its detection is helpful for the diagnosis and treatment of PCa. In addition, the early onset of PCa is dependent on the androgen receptor AR, and its pre-treatment is mainly AR deprivation therapy (ADT) (Tiwari et al., 2020). However, ADT does not cure PCa. Generally, after a median treatment time of 14–30 months, many patients will eventually develop CRPC (Fujita and Nonomura, 2019). Therefore, we also speculate on the expression level of SLC17A9 in CRPC tissues or metastatic tissues. We found that SLC17A9 expression in PCa patients with ERG fusion status and AR amplification status was decreased, manifesting downregulated SLC17A9 expression in PCa patients with ERG fusion status and AR amplification status. These studies showed that SLC17A9 plays a protective role in PCa patients. This result echoes the result of Figure 2B. Next, our experimental results proved that knockdown of SLC17A9 reversed the inhibition of LINC01679 overexpression or miR-3150a-3p inhibitors on cell proliferation, invasion, and migration, and enhancement of cell apoptosis. The above findings confirmed that LINC01679 was the ceRNA that competitively bound to miR-3150a-3p to positively regulate SLC17A9 expression, thereby inhibiting PCa progression.

In conclusion, the present work indicated that LINC01679 was a potential antitumor factor for PCa, which inhibited PCa development by competitively binding to miR-3150a-3p and the mediation of SLC17A9 level. As a result, the present work suggested that the LINC01679/miR-3150a-3p/SLC17A9 axis was possibly related to PCa treatment.

## Data Availability Statement

The datasets presented in this study can be found in online repositories. The names of the repository/repositories and accession number(s) can be found in the article/supplementary material.

## Ethics Statement

The studies involving human participants were reviewed and approved by the Research Ethics Committee of the Affiliated Hospital of Jiangnan University. The patients/participants provided their written informed consent to participate in this study.

## Author Contributions

L-JZ and G-WX participated in the design of this study. Y-YM and L-FZ collected the clinical data. JW and C-YS worked on the analysis and interpretation of data. Y-YM and H-BS performed statistical analysis. Y-YM, L-FZ, and JW conducted experiments in this study. H-BS and FQ collected the background information. Y-YM drafted the manuscript. Y-YM, L-FZ, C-YS, and G-WX provided the funding support. All authors read and approved the final manuscript.

## Conflict of Interest

The authors declare that the research was conducted in the absence of any commercial or financial relationships that could be construed as a potential conflict of interest.

## Publisher’s Note

All claims expressed in this article are solely those of the authors and do not necessarily represent those of their affiliated organizations, or those of the publisher, the editors and the reviewers. Any product that may be evaluated in this article, or claim that may be made by its manufacturer, is not guaranteed or endorsed by the publisher.

## Footnotes

1
https://tcga-data.nci.nih.gov/

2
http://cancergenome.nih.gov/publications/publicationguidelines

3
http://www.targetscan.org/vert_72/

4
http://starbase.sysu.edu.cn/starbase2/index.php

5
http://gepia.cancer-pku.cn/

6
https://www.oncomine.org/resource/login.html

7
http://ualcan.path.uab.edu/

8
http://www.cytoscape.org/

9
https://bibiserv2.cebitec.uni-bielefeld.de/rnahybrid

10
https://www.tbi.univie.ac.at/RNA
